# In-Pixel Temperature Sensors with an Accuracy of ±0.25 °C, a 3σ Variation of ±0.7 °C in the Spatial Domain and a 3σ Variation of ±1 °C in the Temporal Domain

**DOI:** 10.3390/mi11070665

**Published:** 2020-07-08

**Authors:** Accel Abarca, Albert Theuwissen

**Affiliations:** 1 Electronic Instrumentation Lab., Microelectronics, EWI, Delft University of Technology, 2628 CD Delft, The Netherlands; albert@harvestimaging.com; 2 Harvest Imaging, 3960 Bree, Belgium

**Keywords:** in-pixel temperature sensors, Tixel, dark current, CMOS image sensor

## Abstract

This article presents in-pixel (of a CMOS image sensor (CIS)) temperature sensors with improved accuracy in the spatial and the temporal domain. The goal of the temperature sensors is to be used to compensate for dark (current) fixed pattern noise (FPN) during the exposure of the CIS. The temperature sensors are based on substrate parasitic bipolar junction transistor (BJT) and on the nMOS source follower of the pixel. The accuracy of these temperature sensors has been improved in the analog domain by using dynamic element matching (DEM), a temperature independent bias current based on a bandgap reference (BGR) with a temperature independent resistor, correlated double sampling (CDS), and a full BGR bias of the gain amplifier. The accuracy of the bipolar based temperature sensor has been improved to a level of ±0.25 °C, a 3σ variation of ±0.7 °C in the spatial domain, and a 3σ variation of ±1 °C in the temporal domain. In the case of the nMOS based temperature sensor, an accuracy of ±0.45 °C, 3σ variation of ±0.95 °C in the spatial domain, and ±1.4 °C in the temporal domain have been acquired. The temperature range is between −40 °C and 100 °C.

## 1. Introduction

Nowadays, CMOS image sensors are widely used in different applications like astronomy, medicine, and especially in mobile phones [1,2,3]. For many years charge coupled devices (CCDs) dominated the field of image sensors in all kind of related applications. However, the appearance of the active pixel sensor (APS) emerged as a replacement of CCDs [4]. In the last decades, efforts to improve the performance of APS have been made. Currently, the APS has several advantages over CCD like lower cost, lower power consumption, higher dynamic range, and higher integrability [5,6,7]. At the same time, CMOS based temperature sensors are used in many applications like on-chip thermal control, human body temperature monitoring, processor speed, and even in food monitoring [8,9,10]. The dark current or leakage current of the CIS is one of the major contributors of FPN and becomes important under low light condition and high temperature variations. The dark current linearly depends on the integration time of the pixels and exponentially on the temperature variation [11]. In fact, the dark current doubles every ~5–10 °C [12,13,14]. Different techniques are applied to compensate for the dark current. The most common one is to take a dark reference frame at the beginning of the picture acquisition, at a certain exposure time with closed shutter, and then subtracting this dark reference frame from the following images. However, the temperature must be kept constant during the acquisition, otherwise the dark current level changes and a new dark reference frame should be taken. Also, modifications of the photodetector at the physical level have been made to reduce the effect of the dark current. For instance, by adding a p-well layer surrounding the pixel [15], or by using a buried-channel source follower instead of a surface-mode source follower [16].

In our previous works [17,18], the concept of integrating CMOS based temperature sensors has been proved. Nevertheless, our temperature sensors need some improvement to reach a better 3σ variation (spatial and temporal) to compensate for dark current more accurately. In this article, improvements of the in-pixel temperature sensors by using DEM, CDS, BGR bias current and BGR voltage references, and sequential compensation to increase the accuracy of the temperature sensors in a wider temperature range are presented.

This article is organized as follows. Section 2 briefly explains the architecture of the sensor including the temperature sensors. In Section 3, sources of inaccuracy in temperature sensors are discussed. The circuits to overcome the sources of inaccuracy are presented in Section 4. In Section 5, measurement results are presented. A conclusion is given at the end of this paper.

## 2. CMOS Image Sensor with In-Pixel Temperature Sensors

**Figure 1:**
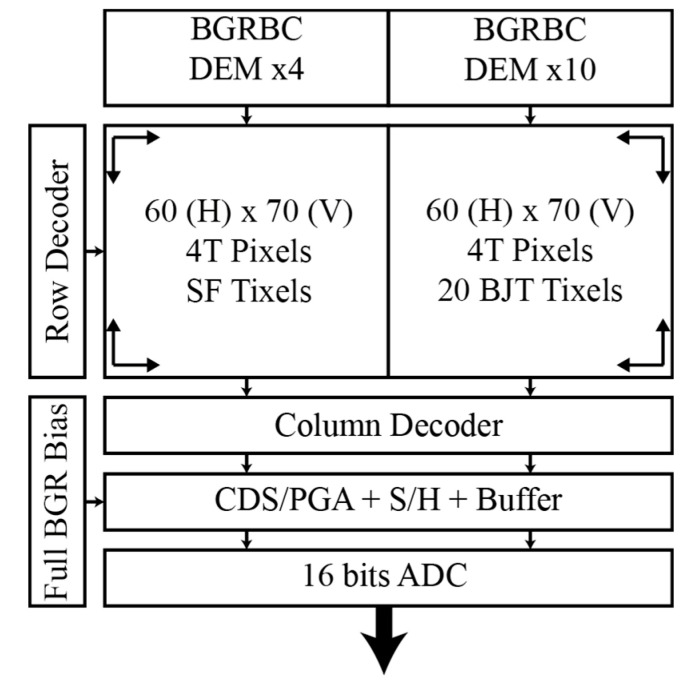
Block diagram of the CMOS image sensor.

The block diagram of the CIS is shown in Figure 1. The sensor is composed of row and column decoders, the pixel array of 60 × 140 pixels, and the readout circuit. Temperature measurements can be performed either by the substrate parasitic bipolar or by the pixel itself via the source follower (SF) transistor. In the pixel array, 20 pixels have been replaced by bipolar temperature sensor pixels (Tixels), and they can perform temperature measurements at the same time as the pixels at the cost of one dead pixel at the position of each Tixel. In the case of the SF temperature sensor, it is the pixel itself performing temperature and video measurements but in different phases incurring in a frame lost. Pixels and temperature sensors use the same readout system composed of a programmable gain amplifier (PGA), a sample and hold (S/H) circuit, an output buffer, and an off-chip 16 bits ADC.

### 2.1. Parasitic Bipolar Temperature Sensor

**Figure 2:**
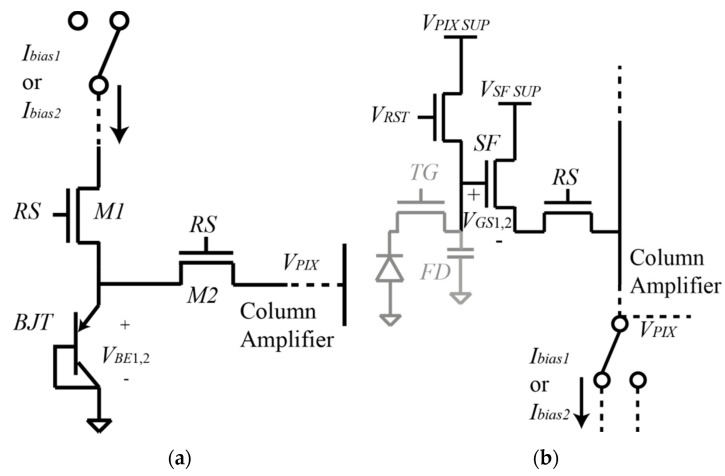
(**a**) Schematic of the parasitic substrate BJT based temperature sensor. (**b**) Schematic of the 4T pixel and the nMOS SF based temperature sensor.

The Tixel is based on a substrate (or vertical) parasitic pnp BJT connected in a common collector configuration. The BJTs are placed in the same layer as the pixels and they (BJT and pixels) share the same readout system. The BJT occupies the area of one pixel: 11 × 11 μm, and its temperature information can be read at the same time as the pixels. Figure 2a shows the schematic of the Tixel.

In Figure 2a, *I_bias_*_1_ and *I_bias_*_2_ correspond to the bias currents of the BJT, and *RS* is the row select signal that controls the switches *M1* and *M2* turning on and off the BJT. The *RS* signal is the same for Tixels and pixels. Comparing this design to the one in [17], the source follower has been deleted because it added extra non-linearity to the BJT output signal.

To avoid mismatch, only one BJT (instead of two) is used as a temperature sensor. The temperature is obtained via the differential base-emitter voltage ($\Delta V_{BE}$) when the BJT is biased by two different currents in a ratio *N*:1 (*I_bias_*_1_ and *I_bias_*_2_, *I_bias_*_2_ = *N*·*I_bias_*_1_). Equation (1) shows the $\Delta V_{BE}$ which is proportional to absolute temperature (PTAT) [19]:

(1) $$\Delta V_{BE}=\frac{kT}{q}\ln\left(N\right)\to T=\frac{\Delta V_{BE}\cdot q}{k\cdot \ln\left(N\right)}$$

where k is the Boltzmann constant, T corresponds to the absolute temperature, and q is the electric charge. The bias currents are generated by an internal DEM current mirror block and the BGR with temperature independent resistors which provides 1 μA (Section 4.2).

### 2.2. nMOS Source Follower Temperature Sensor

The nMOS source follower temperature sensor is based on the pixel itself using the SF transistor as a temperature sensor. The size of the pixel is 11 × 11 µm, and Figure 2b shows the schematic of the nMOS SF temperature sensor based on the typical 4T pixel architecture [20,21].

When the pixel is used as a temperature sensor, the following voltage requirements have to be fulfilled: $V_{RST}>V_{PIX\text{\_}SUP}+V_{TH\text{\_}RST}$, and $V_{PIX}=V_{PIX\text{\_}SUP}-V_{GS}$, where $V_{RST}$ is the gate voltage of the reset transistor, $V_{PIX\;SUP}$ is the pixel voltage supply, $V_{TH\;RST}$ is the threshold voltage of the reset transistor, $V_{PIX}$ is the output voltage of the pixel, and $V_{GS}$ is the gate-source voltage of the source follower transistor. Apart of this, the transfer gate (*TG*) must be switched off to avoid any charge disturbance at the floating diffusion node, and the SF transistor is biased in its subthreshold region where its I-V characteristic follows an exponential-temperature dependent behavior [18]. As in the case of the BJT, the SF temperature sensor is biased (in its subthreshold region) by two different currents in a ratio *N*:1 to obtain the temperature via the differential gate-source voltage ($\Delta V_{GS}$). Equation (2) shows the $\Delta V_{GS}$ which is PTAT:

(2) $$\Delta V_{GS}=n\frac{kT}{q}\ln\left(N\right)\to T=\frac{\Delta V_{GS}\cdot q}{nk\cdot \ln\left(N\right)}$$

where n corresponds to a process parameter. As in the case of the Tixel, the biasing currents are also generated by using the DEM block and the BGR providing a unit current of 1 μA. 

## 3. Non-Linearities Affecting the Temperature Sensors

For both based temperature sensors, non-linearities exist that affect the accuracy of the temperature measurement. In this section, the main sources of inaccuracy are presented.

### 3.1. Sources of Inaccuracies in BJT

As the temperature sensor is formed by only one BJT, it is the base-emitter voltage ($V_{BE}$) the one is measured in every phase to then calculate the PTAT $\Delta V_{BE}$. The $V_{BE}$ is highly influenced by the bias current $I_{bias}$ and the saturation current $I_{S}$, as shown in Equation (3).

(3) $$V_{BE}=\frac{kT}{q}\ln\left(\frac{I_{bias}}{I_{S}}\right)$$

From Equation (3), the influence of $I_{bias}$ and $I_{S}$ is clear, especially because they are both temperature dependent [22,23]. The influence of the $I_{S}$ can be trimmed out on a one-point calibration [23]. The bias current is usually generated from a well-defined bias voltage utilizing a bias resistor, where the resistor is temperature dependent. To avoid the use of a bias resistor, the design reported here uses a bandgap reference with temperature independent resistors to generate a temperature independent bias current (BGRBC). To reduce the mismatch of the $I_{bias}$, one BGRBC circuit is used to supply the DEM current mirrors. The design of the bias current will be presented in Section 4.

**Figure 3:**
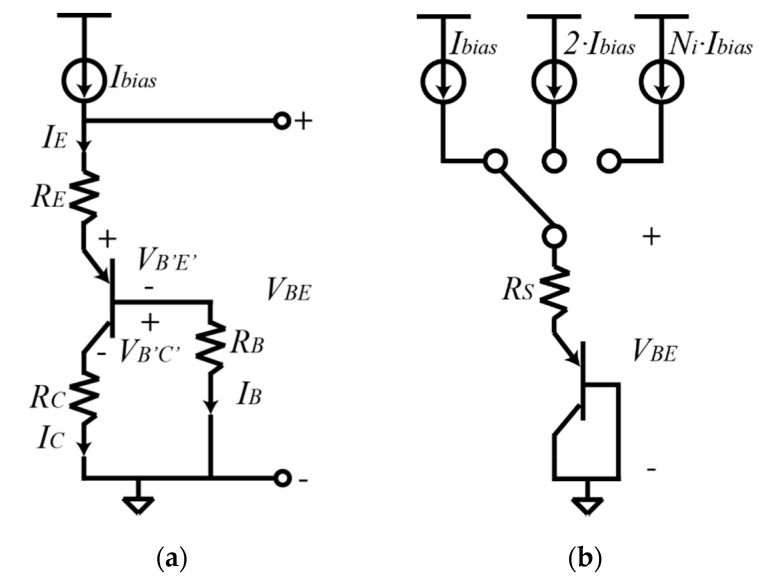
(**a**) Diode connected pnp BJT with series resistances. (**b**) Sequential compensation circuit with effective resistance $R_{S}$.

Another source of inaccuracy is the series resistances of the pnp BJT. Figure 3a shows the diode connected pnp with series resistances.

The external $V_{BE}$ of the BJT including the series resistance is shown in Equation (4):

(4) $$V_{BE}=V_{B^{\prime }E^{\prime }}+I_{E}R_{E}+I_{B}R_{B}=V_{B^{\prime }E^{\prime }}+I_{E}\left(R_{E}+\frac{R_{B}}{\beta_{F}+1}\right)$$

where $V_{B^{\prime }E^{\prime }}$ corresponds to the intrinsic base-emitter voltage, $I_{E}$ is the emitter current, $R_{E}$ is the emitter series resistance, $I_{B}$ is the base current, $R_{B}$ is the base series resistance, and $\beta_{F}$ corresponds to the current gain. The emitter and base resistances can be modelled as a single series resistance $R_{S}$.

(5) $$R_{S}=R_{E}+\frac{R_{B}}{\beta_{F}+1}$$

The effect of the temperature dependent $\beta_{F}$ on $R_{S}$ can be reduced by selecting the right emitter-current (bias current) range where the $\beta_{F}$ is current independent [23]. Therefore, an accurate PTAT differential base-emitter voltage can be generated by a well-defined emitter-current ratio (resulting in a well-defined collector-current ratio as well) where $\beta_{F}$ is independent of the $I_{bias}$ in the selected temperature range. Also, the use of a wide base helps to reduce the non-idealities that result in a current-dependent $\beta_{F}$ (for instance, small diffusion current injected from the base into the emitter.)

Ignoring the effect of the current gain, the base-emitter voltage can be expressed as:

(6) $$V_{BE}=\frac{kT}{q}\ln\left(\frac{I_{bias}}{I_{S}}\right)+I_{bias}R_{S}$$

and the differential base-emitter voltage.

(7) $$\Delta V_{BE}=\frac{kT}{q}\ln\left(N\right)+I_{bias}\left(N-1\right)R_{S}$$

From Equations (6) and (7), it is clear that the bias current and the series resistance affect the linearity of the $V_{BE}$ and $\Delta V_{BE}$. To cancel the effect of $R_{S}$ a sequential compensation is applied by using Equation (8) [23,24]:

(8) $$\frac{N_{i}}{N_{i}-1}V_{BE}\left(I_{bias}\right)-\frac{1}{N_{i}-1}V_{BE}\left(N_{i}\cdot I_{bias}\right)=\frac{kT}{q}\ln\left(\frac{I_{bias}}{I_{S}}\right)-\frac{1}{N_{i}-1}\frac{kT}{q}\ln\left(N_{i}\right)$$

where $N_{i}$ corresponds to the number of bias currents. To generate a $\Delta V_{BE}$ where the $R_{S}$ is cancelled, the BJT is biased by three bias currents, as shown in Figure 3b. For example, if $N_{1}=3$ and $N_{2}=9$, then the differential base-emitter voltage is represented by:

(9) $$4\cdot V_{BE}\left(3I_{bias}\right)-3\cdot V_{BE}\left(I_{bias}\right)-V_{BE}\left(9I_{bias}\right)=\frac{kT}{q}\ln\left(9\right)$$

Equation (9) shows that the series resistance has been cancelled by using sequential compensation. 

### 3.2. Sources of Inaccuracies in nMOS SF

When the nMOS source followerbased temperature sensor is biased in the subthreshold region, the gate-source voltage $V_{GS}$ follows Equation (10) [18]:

(10) $$V_{GS}=n\frac{kT}{q}\ln\left(\frac{I_{bias}}{I_{DS}}\right)-V_{TH}$$

where $I_{DS}$ is the saturation current, and $V_{TH}$ corresponds to the threshold voltage of the source follower transistor. As in the case of the BJT, the bias current and the saturation current have an impact on the value of $V_{GS}$, but also the threshold voltage. The $V_{TH}$ is minimized when the SF is biased with ratiometric currents to obtain the differential gate-source voltage. The variation of $I_{DS}$ leads to a correction of a one-point calibration, while an accurate $I_{bias}$ will be shown in Section 4.2.

A technique to diminish the effect of the factor n, the dimensions W/L of each SF transistor, and the variation of $I_{DS}$ was proposed in [18]. All the pixels in one column are biased to increase the total area of the SF temperature sensor to mimic a larger parallel device with their gates and sources connected to the voltage supply and the output, respectively. A larger device suffers less from process variations. Other consequences of this technique are the reduction of the output noise of the pixel (including thermal and flicker noise). However, this technique has as a drawback that the temperature is not measured locally per pixel but per column. In this paper instead, a large enough bias current has been used to reduce the effect of n and $I_{DS}$ but sufficiently small to not incur in mismatch problems of the SF temperature sensor. This technique was proposed in [25].

### 3.3. Sources of Inaccuracies of the Readout System

The column amplifier suffers from thermal noise because all its components are temperature dependent. One way to compensate for this temperature dependence is to change all the biasing of the amplifier by bandgap references. Another source of inaccuracy is the reference voltage of the amplifier. If the reference is temperature dependent, then the output signal is also affected when the temperature changes. The reference voltage has been changed by an internal BGR. The architecture of the BGR bias voltage and reference voltage is explained in Section 4. 

Also, the column amplifier has an offset produced by the mismatch of the inner transistors that can be cancelled by using CDS. 

## 4. System Design

**Figure 4:**
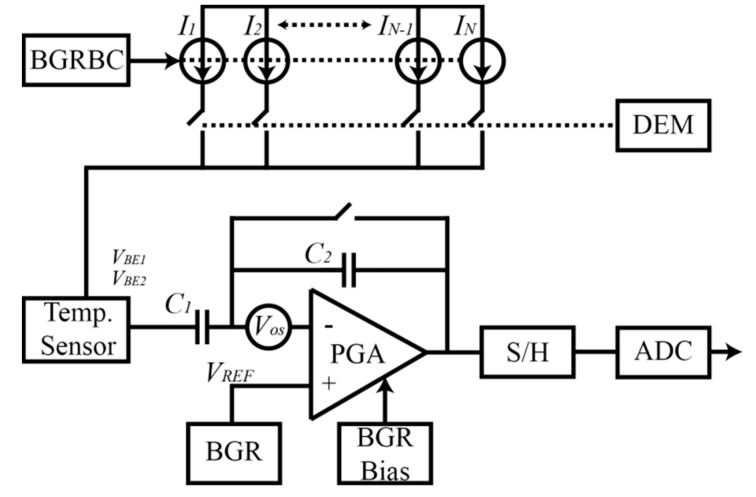
Block diagram of the system.

In our previous work [17], BJTs were integrated into the image array, but no compensation of inaccuracies was made. In [18], compensations for mismatch of the bias currents by using DEM and a larger transistor area of the SF at the bias current level were made. In this work, different techniques have been used to improve the accuracy of the temperature sensors as well as of the readout circuit to decrease the spatial and temporal noise of the system. The mismatch is one of the main components of the spatial noise, while the thermal noise is one of the main components of the temporal noise. The block diagram of the temperature sensor is shown in Figure 4. This block diagram is valid for both types of temperature sensor (BJT and nMOS SF).

The system works as follows: the bandgap reference bias current (BGRBC) provides an almost temperature independent bias current to the temperature sensors to reduce the curvature of $V_{BE}$ and $V_{GS}$, and therefore reduce the error of the temperature sensors. As the temperature sensors (BJT and nMOS SF) measure the temperature via the PTAT differential voltage, they need to be biased by two different currents in a ratio N:1. The current mirror composed of currents *I*_1_ to *I*_N_ provides the current ratio for the temperature sensors. However, due to mismatch, the ratio is not exactly N:1 which leads to an error when comparing different temperature sensors of the same chip. The mismatch is cancelled by using dynamic element matching which averages the total current provided to the temperature sensor. By using DEM, the mismatch error can be reduced by at least one order of magnitude [23]. The output of the temperature sensor is read by a programmable gain amplifier (PGA), where a gain is applied. The PGA suffers from an offset voltage (*V_OS_*) due to the mismatch of the inner transistors of the amplifier. To cancel the *V_OS_*, correlated double sampling (CDS) is applied by using the PGA and the sample and hold (S/H) circuit. Taking the BJT as an example, in one phase, the output voltage $V_{BE1}$ is stored in $C_{1}$ and the offset plus the reference voltage ($V_{OS}+V_{REF}$) are sampled and stored in an analog memory in the S/H circuit. Then, in the next phase the output signal of the temperature sensor $V_{BE2}$ is stored in $C_{1}$, obtaining the differential base-emitter voltage ($\Delta V_{BE}=V_{BE2}-V_{BE1}$) which is amplified by the gain $A=C_{1}/C_{2}$ and stored with ($V_{OS}+V_{REF}$) in another analog memory in the S/H circuit. In this way, subtracting the stored values in both analog memories the offset is cancelled: $V_{out}=\left(A\cdot \Delta V_{BE}+V_{REF}+V_{OS}\right)-\left(V_{REF}+V_{OS}\right)=A\cdot \Delta V_{BE}$.

To reduce the thermal noise of the PGA, bandgap references have been used to bias the PGA and for the reference voltage. In Section 4.3, comparison between using the biasing of [17] and the BGR bias will be shown.

### 4.1. Temperature compensated Resistor

Before explaining the bandgap reference, the resistors that have been used in the BGR will be explained.

In a BGR, resistors are used to set the proper temperature compensation of the output voltage. However, resistors are also temperature dependent and they affect the curvature of the output voltage and the total current. In fact, it is common to not have a temperature independent output voltage and a temperature independent output current at the same time because of the influence of the resistors. 

**Figure 5:**
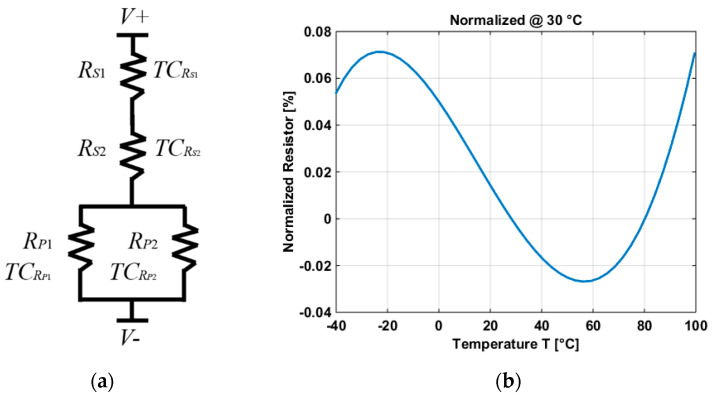
(**a**) Schematic of the temperature independent resistor. (**b**) Variation of the resistor in a temperature range of −40 °C and 100 °C.

A temperature compensated resistor has been designed in such a way to reduce the effect of the temperature dependency of the resistors in the BGR. The resistor is shown in Figure 5a.

The temperature compensated resistor is composed of 4 resistors, two of them in series ($R_{S1}$ and $R_{S2}$) and the other two in parallel ($R_{P1}$ and $R_{P2}$). In both cases (series and parallel), resistors with different and opposite temperature coefficients (TC) have been used and connected to compensate each other. Resistors $R_{S1}$ and $R_{P1}$ are made by using the same highly doped polysilicon with negative TC: $TC_{R_{S1}}$ and $TC_{R_{P1}}$, respectively (where $TC_{R_{S2}}=TC_{R_{P2}}$). Resistors $R_{S2}$ and $R_{P2}$ correspond to the same lowly doped polysilicon resistor with positive TC: $TC_{R_{S2}}$ and $TC_{R_{P2}}$, respectively (where $TC_{R_{S2}}=TC_{R_{P2}}$). The total resistance ($R_{T}$) and the total TC are shown in Equations (11) and (12), respectively:

(11) $$R_{T}=R_{S1}+R_{S2}+\frac{R_{P1}R_{P2}}{R_{P1}+R_{P2}}$$

(12) $$\frac{\partial R_{T}}{\partial T}=0=R_{S1}TC_{R_{S1}}+R_{S2}TC_{R_{S2}}+\frac{R_{P1}R_{P2}\left(R_{P1}TC_{R_{P2}}+R_{P2}TC_{R_{P1}}\right)}{{\left(R_{P1}+R_{P2}\right)}^{2}}$$

Treating the series and the parallel resistors separately, the value of the resistors can be chosen by Equations (13) and (14):

(13) $$\frac{R_{S1}}{R_{S2}}=\frac{TC_{R_{S2}}}{TC_{R_{S1}}}$$

(14) $$\frac{R_{P1}}{R_{P2}}=\frac{TC_{R_{P1}}}{TC_{R_{P2}}}$$

The curvature of the resistors in series is convex and the curvature of the resistors in parallel is concave. Combining both curvatures, the variation of the resistance results in a TC of 0.085 ppm/°C, as shown in Figure 5b.

### 4.2. Bandgap Reference with Temperature Compensated Resistors

**Figure 6:**
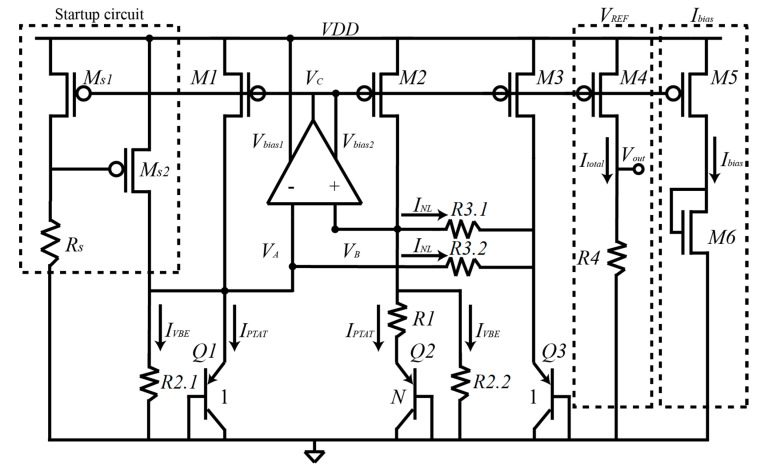
Block diagram of the bandgap reference used to generate temperature independent voltages and temperature independent bias current.

A bandgap reference (BGR) has been used to implement the bias current of the temperature sensors (BJT and nMOS) as well as the bias and the reference of the PGA. The bandgap circuit is based on [26,27] and it is used to generate both the bias current and the biasing of the PGA. The circuit of the BGR is shown in Figure 6.

The BGR of Figure 6 is based on the idea of properly combine currents that compensate each other to generate an almost temperature independent current at the output through the transistors *M4* (for an output voltage) and *M5* (for an output bias current). The BGR works as follows: the OPAMP forces voltages $V_{A}$ and $V_{B}$ to be equal, this results in equal currents through transistors *M1* and *M2*. Transistors *Q2* and *Q1* have an emitter area ratio of *N*, generating a differential base-emitter voltage ($\Delta V_{BE}$) resulting in a PTAT current $I_{PTAT}$ in R1. As resistors R2.1 and R2.2 are nominally identical, a current in R2.1 (and R2.2 ) ($I_{VBE}$) that is proportional to the base emitter-voltage of *Q1* (and *Q2*) is generated. The current $I_{NL}$ is proportional to the differential base-emitter voltage between *Q1* and *Q3* in R3.1 (R3.1=R3.2). Currents $I_{PTAT}$ and $I_{NL}$ are used to compensate first order and higher order non-linearities of $I_{VBE}$, respectively. Therefore, the total current $I_{total}$ in transistor *M4* is (for simplicity R2.1=R2.2=R2, and R3.1=R3.2=R3):

(15) $$I_{total}=I_{VBE}+I_{PTAT}+I_{NL}=\frac{V_{BE}}{R2}+\frac{\Delta V_{BE}}{R1}+\frac{V_{BEQ1}-V_{BEQ3}}{R3}$$

where the value of $I_{total}$ depends on the temperature behavior of resistors R1_3. This is the reason why the temperature compensated resistor of Section 4.1 is used.

This total current is almost temperature independent which leads to a temperature independent output voltage as well. The output voltage $V_{out}$ and its temperature coefficient are shown in Equations (16) and (17), respectively:

(16) $$V_{out}=R4\cdot I_{total}=R4\left(\frac{V_{BEQ1}}{R2}+\frac{\Delta V_{BE}}{R1}+\frac{V_{BEQ1}-V_{BEQ3}}{R3}\right)$$

(17) $$\frac{\partial V_{out}}{\partial T}=0=R4\left(\underset{first\;order}{\underbrace{\frac{1}{R2}\;\frac{\partial V_{BEQ1}}{\partial T}+\frac{1}{R1}\frac{\partial \Delta V_{BE}}{\partial T}}}+\underset{higher\;order}{\underbrace{\frac{1}{R3}\frac{\partial \left(V_{BEQ1}-V_{BEQ3}\right)}{\partial T}}}\right)$$

Treating the first order TC and higher order TC of Equation (17) separately, the first order non-linearity is compensated by properly choosing the values of R1, R2, and N, satisfying Equation (18):

(18) $$\begin{array}{c} 0=\frac{1}{R2}\frac{\partial V_{BEQ1}}{\partial T}+\frac{1}{R1}\frac{\partial \Delta V_{BE}}{\partial T}=\frac{1}{R2}TC_{V_{BEQ1}}+\frac{1}{R1}\frac{k}{q}\ln\left(N\right)\\ ⟹\frac{R2}{R1}\ln\left(N\right)=\frac{q}{k}TC_{V_{BE}}=23.22 \end{array}$$

where $TC_{V_{BEQ1}}$ is the temperature coefficient of $V_{BEQ1}$ being close to –2 mV/°C.

On the other hand, the base-emitter voltage of a bipolar transistor can be also expressed as [28]:

(19) $$V_{BE}\left(T\right)=V_{BG}-\left(V_{BG}-V_{BE0}\right)\frac{T}{T_{0}}-\left(\eta -\alpha \right)V_{T}\ln\left(\frac{T}{T_{0}}\right)$$

where $V_{BG}$ is the bandgap voltage of silicon, $V_{BE0}$ corresponds to the base-emitter voltage at room temperature, $T_{0}$ corresponds to room temperature, η is a temperature constant dependent on technology (for bipolar is around 4), α corresponds to the temperature dependence of the collector current (equal to 1 if the current is PTAT and equal to 0 if the current is temperature independent [26]), and $V_{T}$ is the thermal voltage equal to kT/q. The current $I_{NL}$ can be expressed by using Equation (20):

(20) $$I_{NL}=\frac{V_{BEQ1}-V_{BEQ3}}{R3}=\frac{V_{T}\ln\left(\frac{T}{T_{0}}\right)}{R3}$$

and the value of R3 can be chosen by comparing Equations (16), (19), and using (20) [26]:

(21) $$R3=\frac{R2}{\eta -1}$$

The value of the resistors *R*1, *R*2, and *R*3 can be properly chosen by using Equations (18) and (21). The emitter area ratio of N is equal to 23, R1 = 50 kΩ, R2 = 400 kΩ, η is 4, and R3 = 135 kΩ.

### 4.3. Post-Layout Simulations of the BGR Current and Voltages

**Figure 7:**
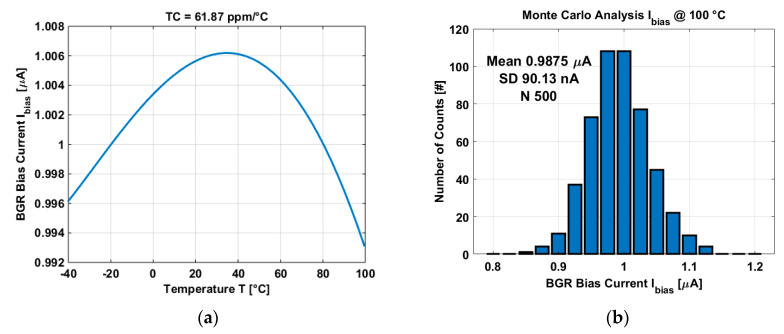
(**a**) Simulation of the BGR bias current of the temperature sensors. (**b**) Monte Carlo analysis of the bandgap reference bias current.

The bias current is generated by properly scaling transistor M5 in the BGR, in this way the bias current is: $I_{bias}=I_{total}/\frac{W}{L}$, where *W/L* is the size of transistor M5. Post-layout simulation shows that the BGRBC provides an almost constant current of 1 µA and an accuracy of 61.87 ppm/°C in the temperature range of −40 °C and 100 °C as shown in Figure 7a.

Figure 7b shows the Monte Carlo analysis of the BGR bias current. The analysis was done by running 500 simulation where the process and mismatch of all components of the BGR where analysed. Results show a very stable BGR with a variation of 9% from the mean value at 100 °C (worst case).

**Figure 8:**
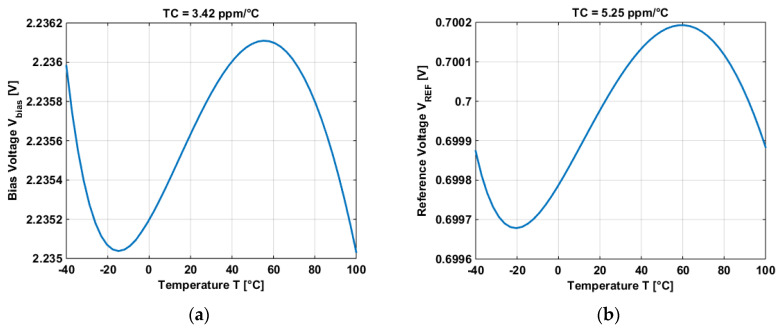
(**a**) Post-layout simulation of one of the bias voltages of the PGA. (**b**) Post-layout simulation of one of the reference voltages of the PGA.

Multiples bias voltages of the PGA have been generated by using the BGR as well as the reference voltage of the PGA. Two different reference voltages have been generated: 0.7 V and 1.1 V. Figure 8 shows one of the bias voltages and one of the reference voltages.

Table 1 shows the different values of the bias voltages and the reference voltages of the PGA.

**Figure 9:**
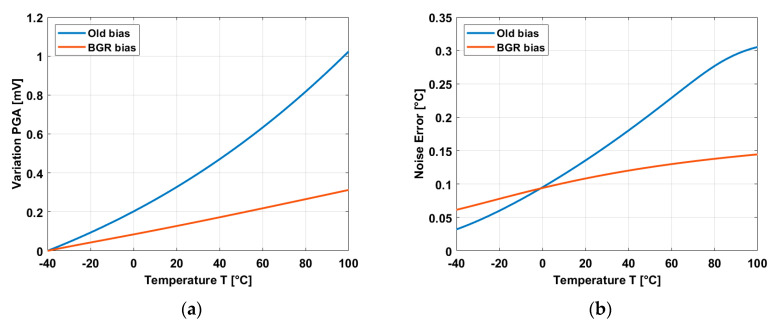
(**a**) Temperature variation of the PGA when it is used in a source follower configuration. (**b**) Noise error of the PGA.

A comparison between using the bias of the PGA in [17] and the bias based on the BGR is shown in Figure 9.

When the PGA is used in a source follower configuration and a constant input voltage is applied, the voltage variation is up to 1 mV by using the old biasing of [17] compared to a variation of 0.3 mV by using the biasing based on BGR, as shown in Figure 9a. If the TC of the temperature sensors is in the order of 1 mV/°C, this means a variation of 1 °C for the old bias and 0.3 °C for the new bias. Figure 9b shows the noise error of the PGA. The thermal noise has been considerable reduced to a maximum of 0.15 °C by using the BGR bias compared to a maximum error of 0.3 °C when the old bias was used. In this way, the temperature dependence of the PGA, which is the main contributor of noise in the readout chain, has been importantly reduced by using a full BGR bias. 

## 5. Measurement Results and Discussion

The sensor was fabricated in a standard CIS 0.18 µm TowerJazz technology. 

The measurement setup is composed of a PCB that provides the main power supplies of the chip, an FPGA generating all control signals, a temperature-controlled oven model VT7004, a reference calibrated temperature sensor Pt-100, and a PC with LabView and Matlab for the processing. To reduce the drift of the oven, the PCB+FPGA are placed in a massive aluminium box, and everything together inside the oven. The temperature of the oven is controlled by a feedback loop with the Pt-100 as a temperature controller.

The measurements were done in a temperature range of −40 °C and 100 °C. For both types of temperature sensors, 100 frames have been taken and averaged when the average accuracy and the 3σ variation in the spatial domain are calculated.

### 5.1. BJT Measurement Results

**Figure 10:**
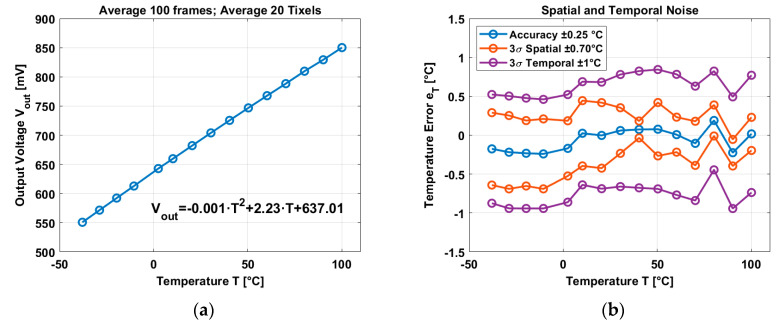
(**a**) Average output voltage in temperature. (**b**) Accuracy, spatial noise, and temporal noise.

The image sensor has 20 Tixels integrated in the array. The results after averaging 100 frames (for the spatial domain) and averaging the 20 Tixels to calculate the accuracy are shown in Figure 10.

Figure 10a shows the average output voltage of the Tixels where the curvature is in the order of 0.1% in the temperature range of −40 °C and 100 °C with a temperature coefficient of 2.23 mV/°C. A 2nd order best curve fitting has been applied to find the accuracy of the bipolar based temperature sensor. An average accuracy of ±0.25 °C has been reached, and the 3σ variation in the spatial domain is ±0.70 °C. The temporal noise has been calculated over 100 frames and it has a 3σ variation of ±1 °C.

### 5.2. nMOS SF Measurement Results

**Figure 11:**
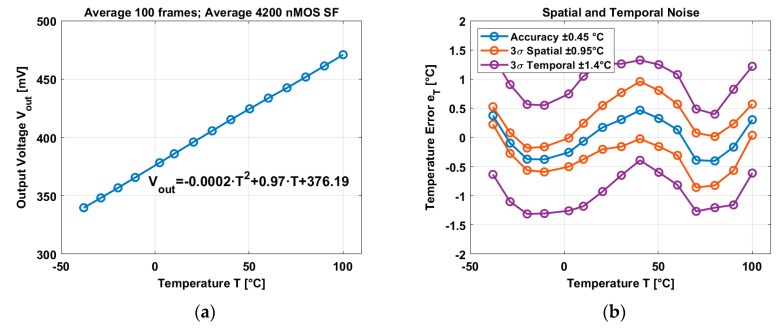
(**a**) Average output voltage in temperature. (**b**) Accuracy, spatial noise, and temporal noise.

The image sensor has 4200 pixels working as image sensor pixels and temperature sensors. Results are shown in Figure 11.

The average output voltage exhibits a curvature in the order of 0.02% and a temperature coefficient of 0.97 mV/°C in the temperature range of −40 °C and 100 °C. The accuracy has been calculated after applying a 2nd order best curve fitting and averaging all the nMOS SF temperature sensors, an accuracy of ±0.45 °C and a 3σ variation of ±0.95 °C have been reached. In terms of the temporal domain, a 3σ variation of ±1.4 °C have been reached.

The results of the temperature sensors (of this paper) have been compared with our previous work in Table 2. The average accuracy as well as the spatial 3σ variation have been improved in a wider temperature range compared to [17] thanks to the use of the different techniques applying in this design. The advantages of the temperature sensors of this work are a small area, good untrimmed average accuracy and spatial 3σ variation, and a wide temperature range.

### 5.3. Dark Current Measurements

**Figure 12:**
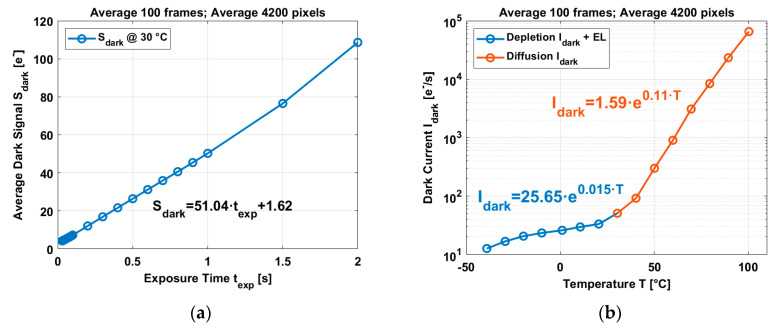
(**a**) Variation of the dark signal with exposure time. (**b**) Variation of the dark current with temperature.

The average dark signal has been measured as a variable of the exposure time where the dark current has been calculated having a value of 51 e^−^/pixel/s at 30 °C, as shown in Figure 12a. The temperature behavior of the dark current exhibits two different curves depending on the temperature range (Figure 12b). It is well known that, for low temperatures, the depletion dark current dominates, while at high temperature, the diffusion dark current takes place [17,30].

In both cases, the dark current follows an exponential behavior, increasing 1.07 times every 5 °C for low temperatures and 1.8 times every 5 °C for high temperatures. At temperatures higher than 25 °C, the CIS shows a typical behavior where the diffusion dark current dominates. However, at temperatures below 20 °C, it seems the depletion dark current is not the dominant mechanism involved because the increment is only 1.07 times per 5 °C instead of ~1.5 times every 5 °C. Moreover, if the dark current at low temperatures is extrapolated by using the data of the diffusion dark current, the values obtained are much lower compared to the measured data. Dark current measurements have shown that pixels next to bipolars exhibit a higher dark current compared to those pixels far from the bipolars. This behavior was observed in [17] and it is related to a possible electroluminescence (EL) effect or charge sharing caused by the bipolars. Thus, the measured dark current at low temperatures might be the result of adding the depletion dark current, and EL (or charge sharing). 

As the output voltage ($V_{out}$) of the temperature sensors is a measure of the temperature, this can be used to predict the dark current, especially at high temperatures. Ignoring the 2nd order term of the temperature sensors, a relation between the dark current (at high temperature) and the temperature sensors is obtained, as shown in Equation (22):

(22) $$\begin{array}{c} I_{dark\text{\_}BJT}=1.59\cdot e^{0.11\left(\frac{V_{outBJT}-637.01}{2.23}\right)}\\ I_{dark\text{\_}nMOS}=1.59\cdot e^{0.11\left(\frac{V_{outnMOS}-376.19}{0.97}\right)} \end{array}$$

## 6. Conclusions

Improvements on the accuracy of the in-pixel temperature sensors have been reached by using a bandgap reference circuit with temperature compensated resistors. The BGR circuit can provide a temperature independent bias current and temperature independent bias/reference voltages by using the same circuit without the need of trimming the circuit. The accuracy of the bias current is 61.87 ppm/°C and the accuracy of the bias/reference voltages is in the order of 4 ppm/°C. The PGA is fully biased by using the BGR circuit, and this results in reducing the thermal noise of the PGA from 0.3 °C to 0.15 °C. Also, the use of DEM to cancel mismatch of the bias current bank, the CDS circuit to cancel the offset of the PGA, and the use of the sequential compensation to reduce the effect of the series resistance have improved the accuracy of the temperature sensors compared to our previous work. The pixels and the temperature sensors have been characterized in a temperature range of −40 °C and 100 °C. The dark current shows two different exponential behaviors with temperature. In the temperature range of −40 °C and 30 °C, the dark current increases 1.07 times every 5 °C and it might be a combination of depletion dark current and electroluminescence (or charge sharing). In the temperature range of 30 °C and 100 °C, the dark current exhibits the typical behavior dominated by the diffusion dark current increasing 1.8 times every 5 °C. This means that the dark current becomes more important for high temperature than for low temperatures. BJT based temperature sensors show an accuracy of ±0.25 °C and a 3σ variation of ±0.7 °C in the whole temperature range, but focusing on the temperature range of 30 °C and 100 °C, the accuracy of the BJTs improves to a 3σ variation of ±0.4 °C. In the case of the nMOS based temperature sensors, they show an accuracy of ±0.45 °C and a 3σ variation of ±0.95 °C. 

## Figures and Tables

**Table 1:** Temperature coefficient and power consumption of the different bias voltages and the reference voltages used in the PGA.

Voltage [V]	TC [ppm/°C]	Power Consumption @ 25 °C [μW]
2.2 (V_Bias_)	3.4239	56
1.1 (V_Bias_)	3.6959	54
0.9 (V_Bias_)	4.3051	52
1.1 (V_REF_)	3.4406	54
0.7 (V_REF_)	5.2471	54

**Table 2:** Comparison with the state-of-art.

Item	[17]	[18]	[29]	This Work	This Work
Year	2018	2020	2018	2020	2020
Process (μm)	0.18	0.18	0.18	0.18	0.18
Type	BJT	nMOS	BJT	BJT	nMOS
Area (µm^2^)	8712 ^1^	121	121	8591 ^1^	8591 ^1^
Power (μW)	15	36	36	33	20
Range (°C)	−40 to 90	−20 to 80	−20 to 80	−40 to 100	−40 to 100
Accuracy (°C)	±0.6	±0.3	±0.5	±0.25	±0.45
Spatial 3σ (°C)	±4	±1.3	±1.1	±0.7	±0.95
Time 3σ (°C)	-	-	-	±1	±1.4

^1^ Including readout system area.
